# Secreted Phosphoprotein 1 Expression in Retinal Mononuclear Phagocytes Links Murine to Human Choroidal Neovascularization

**DOI:** 10.3389/fcell.2020.618598

**Published:** 2021-01-28

**Authors:** Anja Schlecht, Peipei Zhang, Julian Wolf, Adrian Thien, Dennis-Dominik Rosmus, Stefaniya Boneva, Günther Schlunck, Clemens Lange, Peter Wieghofer

**Affiliations:** ^1^Eye Center, Medical Center, Medical Faculty, University of Freiburg, Freiburg, Germany; ^2^Institute of Anatomy, Leipzig University, Leipzig, Germany

**Keywords:** AMD, CNV, Osteopontin, OPN, SPP1, microglia, *Cx3cr1*^*CreERT*2^

## Abstract

Age-related macular degeneration (AMD) represents the most common cause of blindness in the elderly in the Western world. An impairment of the outer blood-retina barrier and a localized inflammatory microenvironment cause sprouting of choroidal neovascular membranes (CNV) in neovascular AMD that are in intimate contact with surrounding myeloid cells, such as retinal microglia, and ultimately lead to visual impairment. The discovery of novel target molecules to interfere with angiogenesis and inflammation is vital for future treatment approaches in AMD patients. To explore the transcriptional profile and the function of retinal microglia at sites of CNV, we performed a comprehensive RNA-seq analysis of retinal microglia in the mouse model of laser-induced choroidal neovascularization (mCNV). Here, we identified the angiogenic factor Osteopontin (*Opn*), also known as “secreted phosphoprotein 1” (*Spp1*), as one of the most highly expressed genes in retinal microglia in the course of CNV formation. We confirmed the presence of SPP1 at the lesion site in recruited retinal microglia in *Cx3cr1*^CreER^:*Rosa26-tdTomato* reporter mice by confocal microscopy and in whole retinal tissue lysates by ELISA highlighting a massive local production of SPP1. Inhibition of SPP1 by intravitreal injection of an anti-SPP1 antibody significantly increased the lesion size compared to IgG-treated control eyes. In line with our results in rodents, we found an increased *SPP1* mRNA expression in surgically extracted human choroidal neovascular (hCNV) membranes by the quantitative RNA-seq approach of massive analysis of cDNA ends (MACE). Numerous IBA1^+^SPP1^+^ myeloid cells were detected in human CNV membranes. Taken together, these results highlight the importance of SPP1 in the formation of CNV and potentially offer new opportunities for therapeutic intervention by modulating the SPP1 pathway.

## Highlights

- *Spp1* emerges as one of the top differentially regulated angiogenic genes in murine microglia in the model of laser-induced CNV.
- SPP1 protein expression is highly increased in CNV tissue and present in retinal microglia.
- Scavenging of SPP1 by an anti-SPP1 antibody leads to increased lesion size in CNV.
- SPP1 expression is highly induced in surgically extracted human choroidal neovascularization membranes on both RNA and protein levels.

## Introduction

Microglia represent the resident tissue-macrophages of the retina and the brain and originate from the extra-embryonic yolk sac early during development (Ginhoux et al., 2010; Kierdorf et al., 2013; Goldmann et al., 2016; O'Koren et al., 2019; Wieghofer et al., in press). In the context of neurodegeneration, neuroinflammation, or other insults, the composition as well as the gene and protein expression signatures of myeloid cells can dramatically change (Ajami et al., 2018; O'Koren et al., 2019; Wieghofer et al., in press). These changes include microglia activation leading to relevant functional alterations. The mode of action can thereby be beneficial but also detrimental depending on the disease model affecting microglia in the brain or retina (Reyes et al., 2017; Masuda et al., 2020).

Choroidal neovascularisation (CNV) is a common cause of irreversible vision loss in patients with age-related macular degeneration (AMD), which is the leading cause of blindness in the elderly (Fine et al., 2000). In a previous study, we showed that myeloid cells represent a heterogeneous cell population that accumulates at sites of CNV and modulates its formation in a laser-induced CNV mouse model, which is a widely used model for nAMD (Lambert et al., 2013; Wieghofer et al., in press). The close interplay between myeloid cells and blood vessel formation has been extensively studied in the past; however, the origin of accumulating myeloid cells in this model has long remained unclear (Oh et al., 1999; Fantin et al., 2010; Dejda et al., 2016; Usui-Ouchi et al., 2020). Recently we have shown that retinal microglia are the dominant innate immune cell population at sites of CNV and are characterized by a specific disease-associated gene expression signature similar to other disease models, including *Spp1* encoding the secreted phosphoprotein 1 (SPP1), *Lgals3* (lectin, galactose binding, soluble 3), and *Apoe* (Apolipoprotein E) (Keren-Shaul et al., 2017; O'Koren et al., 2019; Wieghofer et al., in press). In particular, the role of secreted SPP1 in vascular diseases and its potential to serve as an easily accessible biomarker present in blood serum and other body fluids has gained attention recently (Lok and Lyle, 2019). SPP1 is a multifaceted protein involved in homeostatic functions and pathophysiological processes like bone morphogenesis, vascular remodeling, recruitment of leukocytes, cell adhesion, and extracellullar matrix remodeling (Lok and Lyle, 2019). The broad spectrum of features is reflected by the cell types expressing SPP1 including leukocytes, epithelial and endothelial cells, and neurons in humans (Kunii et al., 2009).

As a matricellular cytokine SPP1 binds to integrin receptors, like α_V_ integrins, and certain splice variants of the hyaluronic acid receptor CD44, which are expressed by endothelial cells (Lok and Lyle, 2019). The angiogenic capacity combined with its potential to shape myeloid cell recruitment highlights SPP1 as a promising target in vascular diseases (Yu et al., 2017; Lok and Lyle, 2019).

The goal of our study was to explore gene expression signatures of native and CNV-associated retinal microglia by comprehensive bulk RNA-seq. Furthermore, we investigated the role of SPP1 in CNV formation by local intraocular application of an antibody directed against SPP1. Finally, we correlated our findings in mice to human CNV samples from nAMD patients. Our results underline the importance of SPP1 in the formation of CNV in mice and humans, thus paving the way for new therapeutic interventions by modulating the SPP1 pathway.

## Results

### Identification of the Angiogenic *Spp1* Gene in Experimental Choroidal Neovascularization

**Figure 1:**
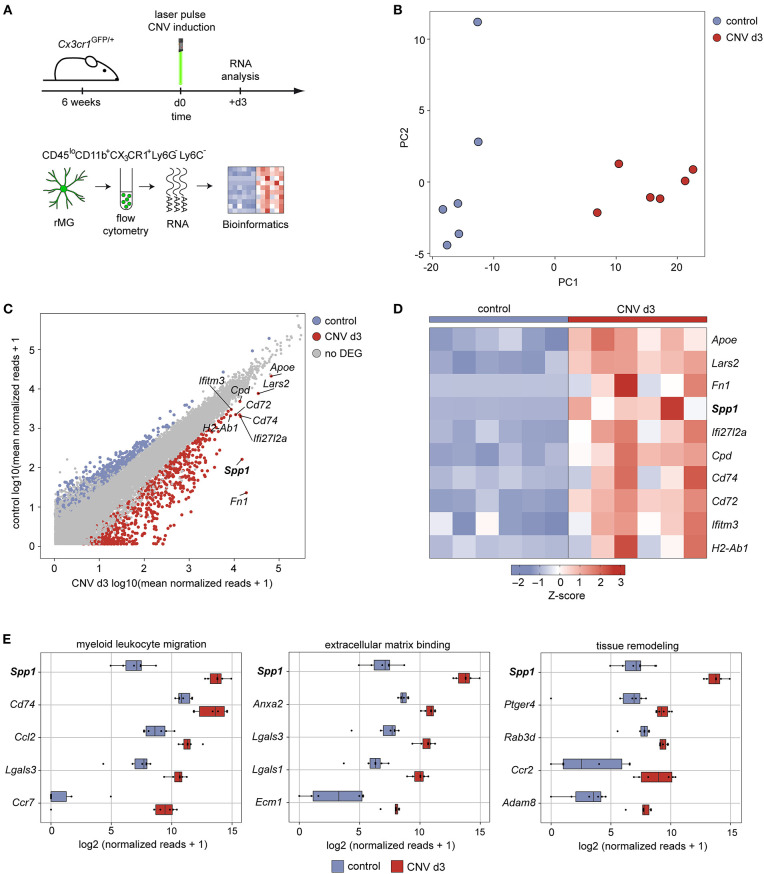
Expression of secreted phosphoprotein 1 increases upon experimental induction of choroidal neovascularization in mice. **(A)** Experimental scheme depicting the workflow for isolation of viable and single CD45^lo^CD11b^+^CX_3_CR1^+^Ly6C^−^Ly6G^−^ cells from the retina under healthy conditions and experimental choroidal neovascularization at d3 after induction for unbiased RNA-seq. Six focal argon laser burns were applied to each retina to induce microglia activation and subsequent choroidal neovascularization formation in mice (mCNV). **(B)** Principal component analysis of transcripts analyzed by RNA-seq. **(C)** Scatter plot using the log2 transformation of normalized counts visualizing differentially expressed genes between healthy conditions (y-axis) and experimental choroidal neovascularization on d3 after laser treatment (x-axis). The top 10 highest expressed genes are labeled. Definition of differentially expressed genes: abs(log2FC) > 1.5 & padjusted < 0.05. **(D)** Heatmap of the top 10 differentially expressed genes between healthy conditions and experimental choroidal neovascularization on d3 after laser treatment according to the mean expression in the laser group. The z-score represents the gene expression in relation to its mean expression by standard deviation units (red: upregulation, blue: downregulation). **(E)** Box plots illustrating the top five factors of the most disease-relevant GO terms ordered according to the mean expression in the laser group.

To gain more insight into the transcriptional profile and angiogenic capacity of microglia during development of experimental choroidal neovascularization, we conducted bulk RNA-seq of flow cytometry-isolated CD45^lo^CD11b^+^CX_3_CR1^+^Ly6C^−^Ly6G^−^ retinal microglia at CNV d3 (Figure 1A). The principal component analysis clearly showed distinct gene expression patterns between the lasered group 3 days after experimental laser treatment and unlasered control mice (Figure 1B). Next, we determined the differentially expressed genes (DEG) between CNV-associated MG and control microglia and found that 654 DEG were significantly increased in microglia in association with CNV formation. Among the top five DEG increased in CNV-associated MG were *Fn1* (log2FC = 9.8, padj. = 2.9 × 10^−15^), *Spp1* (log2FC = 6.6, padj = 3.8 × 10^−25^), *Ifi27I2a* (log2FC = 2.9, padj = 7.7 × 10^−8^), *Cd74* (log2FC = 2.7, padj = 7.9 × 10^−7^), and *Cd72* (log2FC= 2.4, padj = 2.2 × 10^−24^) (Figures 1C,D). Also *Apoe* (log2FC = 1.7, padj = 3.3 × 10^−12^) was significantly upregulated and is an important regulator for the transition into disease-associated microglia (Song and Colonna, 2018). In addition, genes typically expressed by antigen-presenting cells, such as *H2-Ab1* (log2FC = 2.1, padj = 9.3 × 10^−6^) and *Cd74*, or involved in interferon signaling, like *Ifitm3* (log2FC = 1.59, padj = 0.005) and *Ifi27I2a*, were present among the upregulated genes in CNV (Figures 1C,D). To identify functionally associated genes, we performed a gene ontology (GO) analysis and found genes relevant for the biological processes “myeloid leukocyte migration,” “extracellular matrix binding,” and “tissue remodeling” (Figure 1E). These genes include *Ccl2* (log2FC = 2.5, padj = 4.2 × 10^−7^) encoding a chemokine attracting peripheral blood monocytes and *Lgals3* (log2FC = 3.1, padj = 8.2 × 10^−10^) that has been described in disease-associated microglia and is responsible for interaction with the extracellular matrix (O'Koren et al., 2019; Boeck et al., 2020) (Figure 1D). Nevertheless, besides its known role in angiogenesis, *Spp1* was also the most highly expressed gene within all three biological processes GO terms, which prompted us to investigate its role in further detail (Figure 1E).

### Validation and Interference With SPP1 on the Protein Level in CNV Mice

**Figure 2:**
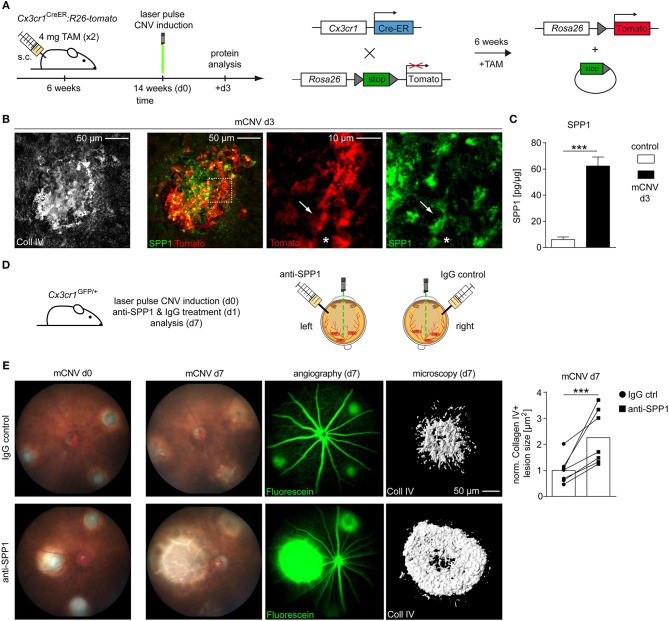
Interference with SPP1 on protein level changes the outcome of experimental choroidal neovascularization. **(A)** Experimental scheme. Six weeks old *Cx3cr1*^*CreER*^*:Rosa26-tdTomato* mice were exposed to TAM to induce microglia-specific Tomato labeling. Eight weeks later three focal argon laser burns were applied to each retina to induce microglia activation and subsequent choroidal neovascularization (mCNV). Mice were sacrificed at CNV d3 for immunofluorescence stainings. **(B)** Representative immunofluorescence images for collagen IV (white), Tomato (red), and SPP1 (green) in *Cx3cr1*^*CreER*^*:Rosa26-tdTomato* mice on d3 after CNV induction. Arrows point to a Tomato^+^SPP1^+^ rMG while asterisks indicate a Tomato^+^SPP1^−^ rMG. Representative pictures of seven mice out of two independent experiments are displayed. **(C)** SPP1 levels in lesioned RPE tissue (mCNV) of wildtype mice compared to untreated controls d3 after CNV induction as measured by ELISA (control: *n* = 7, CNV: *n* = 8 from two independent experiments). Bars represent means ± s.e.m. ***p < 0.0001, Unpaired *t*-test. **(D)** Experimental scheme. Choroidal neovascularization was laser induced at day 0 (d0) in *Cx3cr1*^*GFP*/+^ mice. At d1, 50 ng anti-SPP1 (dissolved in 1 μL PBS injection volume) was injected intravitreally into one eye and the same amount of IgG control antibody (again dissolved in PBS) into the contralateral eye. Mice were sacrificed at d7 after evaluation of fundus and fluorescence angiography. **(E)** Exemplary funduscopic image of lesions directly after laser burn-induced mCNV at day 0 (left) and d7 (right) under treatment with IgG control (top row) or anti-SPP1 antibody (bottom row). Fluorescence angiography indicates leakage of intraperitoneally applied Fluorescein. Representative Imaris reconstruction of collagen IV immunoreactivity and corresponding quantification of CNV size in mice that underwent laser treatment and subsequent injection of either anti-SPP1 into the left eye (squares) or IgG isotype controls into the right eye (circles). Each symbol represents the mean lesion size within one eye of one mouse, based on Collagen IV area of individual lesions imaged by confocal microscopy and normalized to the IgG control. Corresponding values from left and right eye are displayed as pairs. Bar graphs represent the overall mean value of seven mice in total. All values were normalized to the IgG control mean value. Bars represent mean ± s.e.m. ***p < 0.001, ratio paired *t*-test.

To validate the expression of SPP1 protein we used conditional *Cx3cr1*^*CreER*^*:Rosa26-tdTomato* reporter mice to specifically label microglia in the CNV model (Goldmann et al., 2013; Wieghofer et al., 2015, in press). Eight weeks after Tamoxifen treatment to induce Tomato expression in retinal microglia, mice were laser-treated to induce CNV (d0) and sacrificed 3 days later for immunofluorescent staining and confocal imaging (Figures 2A,B). Here, we found numerous Tomato^+^ microglia cells expressing SPP1 and exhibiting an amoeboid appearance, which indicates an activated phenotype in contrast to their ramified state under homeostatic conditions (Figure 2B). Tomato^−^SPP1^+^ cells were present indicating that other cell types than microglia are also involved in CNV formation. In accordance with our RNA-seq data, a strong increase of SPP1 protein in whole CNV tissue was detectable by ELISA (CNV: 62.30 ± 6.90, control: 6.00 ± 2.03, *p* < 0.0001) (Figure 2C). These findings prompted us to inhibit SPP1 in the course of CNV development by an intravitreal administration of an anti-SPP1 antibody 1 day after CNV laser-induction. The contralateral eyes were treated with an IgG control (Figure 2D). The increase in lesion size was clearly visible over time by funduscopic imaging and showed severely enhanced vascular leakage under anti-SPP1 treatment visualized by angiography (Figure 2E). The quantification of the lesion size based on Collagen IV immunoreactivity revealed a more than 2-fold increase of the lesioned area (2.25 ± 1.05, *p* = 0.0004) in eyes treated with anti-SPP1 compared to IgG controls. Consequently, the intraocular application of anti-SPP1 antibodies suggested a protective, anti-angiogenic function of SPP1 during CNV formation (Figure 2E).

### SPP1 Expression in Mice Can Be Linked to Human Neovascular AMD

**Figure 3:**
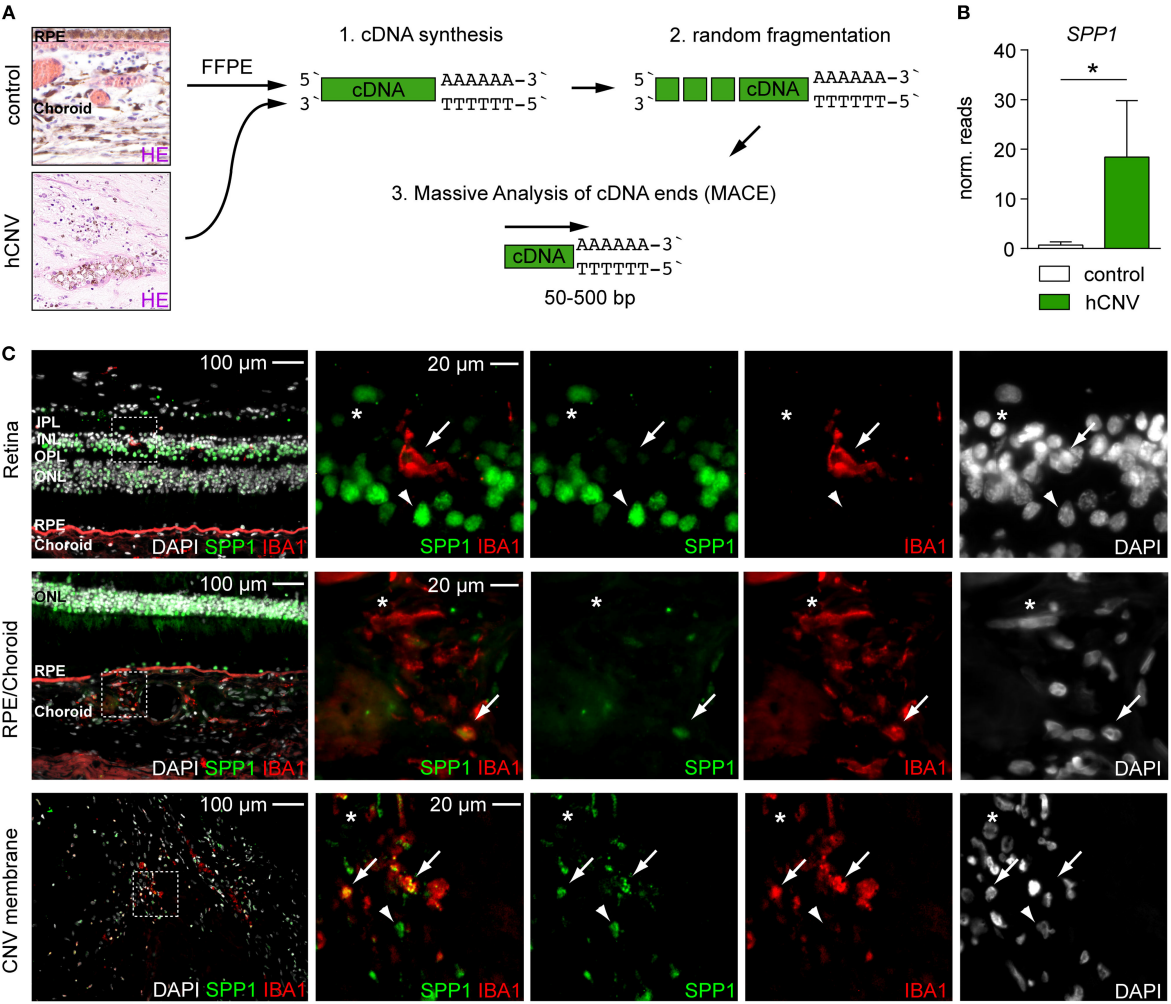
Quantitative RNA-seq analysis and immunofluorescence reveal the presence of SPP1 in human CNV tissue. **(A)** Experimental scheme. RNA was extracted from formalin-fixed and paraffin-embedded human healthy choroid and CNV membranes for massive analysis of cDNA ends (MACE) RNA-seq analysis. Retinal pigment epithelium is abbreviated with RPE. **(B)** *SPP1* expression was quantified by RNA-seq (MACE) in four control samples and four CNV membranes (*p* = 0.019). Bars represent mean ± s.e.m. **(C)** Immunofluorescence for DAPI (white), SPP1 (green), and IBA1 (red) in the retina and RPE/choroid of one body donor and in a CNV membrane of a patient diagnosed with neovascular age-related macular degeneration. IBA1^+^SPP1^+^ macrophages (arrow) could be found in both healthy RPE/choroid and CNV membranes in line with the RNA-seq (MACE) results, next to IBA1^+^SPP1^−^ macrophages (asterisk). Arrowheads point to IBA1^−^SPP1^+^ cells. Dashed white squares indicate the region of interest shown as magnifications on the right side. The layering is indicated for the inner and outer plexiform layer (IPL, OPL), inner and outer nuclear layer (INL, ONL), retinal pigment epithelium (RPE), and choroid.

To generalize our findings, we next explored the expression of SPP1 in human CNV by conducting a *post-hoc* analysis of already available RNA-seq data from surgically extracted human membranes of choroidal neovascularization (hCNV) (Figure 3A) (Schlecht et al., 2020). Interestingly, also in human CNV, *SPP1* was one of the most prominent upregulated angiogenic genes when compared to healthy controls (CNV: 18.41 ± 11.38 reads, controls: 0.70 ± 0.64 reads, *padj* = 0.019) (Figure 3B). Next, we investigated the expression of SPP1 protein in human body donor tissue as compared to human CNV membranes by immunofluorescence (Figure 3C). In retinal IBA1^+^ microglia, SPP1 was barely detectable in healthy control tissue, which is in line with previous studies (Masuda et al., 2019) (Figure 3C). Only in the choroid, a faint staining for SPP1 was observed in IBA1^+^ macrophages, whereas in CNV tissue a strong expression of SPP1 was detected in IBA1^+^ cells. Non-myeloid IBA1^−^SPP1^+^ cells were also present at the site of the lesion, similar to our findings in mice (Figures 2B, 3C). Overall, we found comparable expression of *SPP1* RNA and SPP1 protein in both murine and human tissue samples, suggesting a functional relevance of the molecule in CNV formation.

## Discussion

Retinal microglia cells are key players in the modulation of disease course and severity across disease models including retinal degeneration, diabetic retinopathy, retinal light damage, and CNV (O'Koren et al., 2019; Van Hove et al., 2020; Wieghofer et al., in press). The concept of disease-associated microglia (DAM), initially introduced for brain disease models involving microglia activation, was recently successfully applied to retinal microglia (Keren-Shaul et al., 2017; O'Koren et al., 2019; Wieghofer et al., in press).

Single-cell RNA-seq (scRNA-seq) of fresh tissue samples is advantageous to characterize subtypes of all kinds of cell types. However, fresh human CNV tissue is not available as surgical CNV extraction became obsolete due to novel treatment strategies and archived samples are not amenable to scRNA-seq preparation (Masuda et al., 2019, 2020; Schlecht et al., 2020). To compare gene expression signatures across species, we applied bulk RNA-seq approaches on experimentally induced murine and archived human choroidal neovascularization tissue samples.

Gene expression profiling of retinal microglia by RNA-seq revealed a CNV-associated phenotype clearly distinguishable from the profile of retinal microglia in untreated control mice. Significant changes were observed for transcripts functionally relevant in fibrosis (*Fn1*), antigen-presentation (*Cd74, H2-Ab1*), myeloid cell migration (*Cd74, Ccl2, Lgals3*), phagocytosis (*Lgals3*), and inflammation (*Ifi27I2a, Ifitm3, Lgals3*). With regard to antigen presentation, we recently demonstrated that *Cd74* is not expressed in retinal microglia under homeostatic conditions but induced together with *H2-Aa*, encoding MHCII, upon CNV induction, which is in line with the present study (Wieghofer et al., in press). Of note, the induced expression of MHCII (*H2-Aa*) was also confirmed on the protein level in mice and in human CNV membranes (Wieghofer et al., in press). The observed increase of *Apoe* expression further supports the induction of the DAM gene expression signature mediated by binding to the triggering receptor of myeloid cells 2 (TREM2), like it was shown in the brain (Song and Colonna, 2018). Furthermore, *Apoe* was highly expressed in DAM in a model of retinal light damage and, most importantly, in mononuclear phagocytes in the subretinal space in AMD patients (Levy et al., 2015a,b; O'Koren et al., 2019). In another study, SPP1 was found to be key for the promotion of macrophage survival in the subretinal space in the CNV model (Beguier et al., 2020).

The highly significant induction of *Spp1* in retinal microglia points to this gene as a key mediator of CNV pathology, which could be functionally associated with three major hallmarks of the CNV model: angiogenesis, macrophage recruitment, and tissue remodeling of the extracellular matrix (Tobe et al., 1998; Lambert et al., 2013). Also in a murine model of light-induced neurodegeneration, *Spp1* was highly induced in a specific microglial cluster that also expressed *Lgals1* and *Lgals3* as distinctive marker for subretinal DAM (O'Koren et al., 2019).

In line with previous CNV studies, the lesion size was affected by varying degrees in *Spp1*^−/−^ mice or after systemic application of an antibody directed against SPP1 (Fujita et al., 2011; Ong et al., 2011; Beguier et al., 2020). In these experiments, which are based on systemic inhibition of SPP1, CNV size is decreased (Fujita et al., 2011; Ong et al., 2011), whereas in the current study, local inhibition of SPP1 by antibodies leads to larger lesions. This can be explained by several factors. The systemic inhibition of SPP1 not only leads to smaller lesions but also to severe changes in the hematopoiesis of myeloid cells in the bone marrow, which, as previously shown, is reflected by a reduced number of the monocyte-macrophage dendritic cell progenitors (MDP) (Magdaleno et al., 2018). A systemic treatment could thereby alter the overall composition of myeloid cells in the blood, finally leading to lower numbers of recruited macrophages and smaller lesions (Fujita et al., 2011). In the clinical routine an antibody against vascular endothelial growth factor (VEGF) is applied intravitreally in patients suffering from neovascular AMD to achieve high local concentration and reduce the possibility of side effects. Therefore, we administered anti-SPP1 in a more specific approach by applying it locally into the eye to minimize its influence on peripheral immune compartments. In a recent study, the same antibody was successfully applied *in vivo* proving its suitability and bioactivity after intravitreal injection (Beguier et al., 2020) besides its use *in vitro* (Hosaka et al., 2017; Hulsmans et al., 2018). Despite the same mode of application in the CNV model, anti-SPP1 was only given in combination with other factors, and the significantly differing treatment regimen with respect to dosage, time points, and frequency of applications together with a later time point of analysis after CNV does not allow a direct comparison (Beguier et al., 2020). In contrast to previous studies including CNV experiments in constitutive *Spp1*^−/−^ mice, we found a more than 2-fold increase in lesion size at CNV d7 with anti-SPP1 treatment (Ong et al., 2011). Our findings support a protective function of SPP1 during CNV development that could be mediated at the level of myeloid cells but also through other sources of SPP1 (Lok and Lyle, 2019). Several studies point toward a microglia-related effect. First, this is supported by the fact that microglia were shown to be the predominant myeloid cell type at the lesion site (Wieghofer et al., in press) and their expression of SPP1 upon CNV induction, as confirmed on RNA and protein levels. Furthermore, treatment with SPP1 *in vitro* revealed functionally relevant changes in microglia response to external stimuli (Tambuyzer et al., 2012; Patouraux et al., 2014; Rabenstein et al., 2016). In LPS-stimulated microglia, the production of pro-inflammatory cytokines, like IL-6 and TNFα, was significantly reduced under SPP1 treatment, thereby promoting an anti-inflammatory phenotype (Rabenstein et al., 2016). In addition, the presence of SPP1 significantly improved the survival of microglia under stress conditions and increased the phagocytic activity of microglia *in vitro* (Tambuyzer et al., 2012; Rabenstein et al., 2016). A dose-dependent decrease in superoxide production was found, as well, which is in line with an increase of iNOS expression upon SPP1 decrease in a macrophage cell line (Tambuyzer et al., 2012; Patouraux et al., 2014). In the same study, the authors could reverse the SPP1-mediated increased proliferation rate of microglia by applying an anti-OPN antibody *in vitro* (Tambuyzer et al., 2012), which resembles our antibody-mediated interference with SPP1 in the experimental CNV model. Of note, secreted SPP1 stimulates macrophages to produce CCL2, which was also detected in CNV-associated retinal microglia and acts as a potent chemokine for the recruitment of peripheral myeloid cells (Rowe et al., 2014). These cells could further modulate angiogenesis but represent only a fraction of the overall myeloid cell composition dominated by microglia (Wieghofer et al., in press). However, if the aforementioned *in vitro* observations in microglial biology would apply to the *in vivo* situation, the blockage of SPP1 would have an escalating effect on the basic functions of microglia, like pro-inflammatory cytokine and production of reactive oxygen species, leading to a worsening of tissue damage at the lesion site. The availability of conditional CreERT2 deleter strains targeting microglia like the *Cx3cr1*^CreER^ mouse model opens not only the possibility to label microglia specifically but also create conditional knockout mice (Droho et al., 2020; Wieghofer et al., in press). This strategy was successfully applied in the past and will decipher microglial functions in a much more specific setting in the future (Lückoff et al., 2016; Wolf et al., 2020).

Beside the observed role of microglia, we cannot completely rule out a functionally relevant effect of the anti-SPP1 treatment on other non-myeloid cells. For example, SPP1 is also expressed by endothelial and smooth muscle cells and loss of stroma-derived SPP1 led to enhanced tumor progression in mice (O'Brien et al., 1994; Szulzewsky et al., 2017). Interestingly, the interaction of a CD44v6 splice variant, acting as a receptor for SPP1, with VEGF receptor 2 (VEGFR2) on endothelial cells suggests an interference with SPP1 signaling across cell types (Tremmel et al., 2009). Furthermore, our study shows a considerable expression of SPP1 in human retinal neurons, which is consistent with findings in rat retina (Chidlow et al., 2008). Thus, an indirect effect of anti-SPP1 treatment via modulation of the SPP1 pathway in neurons cannot be excluded. RNA-seq analysis of whole human CNV membranes revealed *SPP1* as one of the most highly regulated genes within the disease-relevant GO terms “extracellular structure organization” and “response to wounding,” similar to our findings in mice (Schlecht et al., 2020). Our immunofluorescent analysis revealed that SPP1 was predominantly expressed in IBA1^+^ mononuclear phagocytes in human CNV, which strongly resembled the situation present in the murine experimental CNV model, suggesting a highly conserved phenomenon across species. Given the increasing expression of SPP1 in human microglia with age (Sankowski et al., 2019), the SPP1 signaling pathway in MG may modulate CNV development in patients with neovascular AMD and therefore represent a therapeutic target in the future.

Taken together, this study shows that Spp1 is significantly expressed at RNA and protein level in murine and human CNV. The intravitreal application of anti-SPP1 antibodies led to increased CNV lesion size suggesting an anti-angiogenic effect of SPP1. Our results provide new insights into the biology of retinal microglia during health and CNV formation and open new avenues for the treatment of ophthalmological diseases like neovascular AMD.

## Materials and Methods

### Mice

In this study, C57BL/6J mice were used as wildtype (WT) mice. All transgenic lines [*Cx3cr1*^*GFP*/+^, *Cx3cr1*^*CreERT*2^, and *Rosa26-fl-stop-fl*-t*dTomato* (*Rosa26-tdTomato*)] were bred on a C57BL/6J background under specific pathogen-free conditions and devoid of *Crb1* (*RD8*) mutations. *Cx3cr1*^*CreERT*2^ were crossed to *Rosa26-tdTomato*. All animal experiments were approved by local administration and were performed in accordance with the respective national, federal, and institutional regulations.

### Tamoxifen Treatment

For induction of the nuclear CreER-T2 recombinase activity in adult animals, 6–8-week-old *Cx3cr1*^*CreER*^ mice were treated with 4 mg Tamoxifen (TAM, T5648-1G, Sigma-Aldrich, Taufkirchen, Germany) dissolved in 200 μl corn oil (Sigma-Aldrich, C8267-500 ml) and injected subcutaneously at two time points 48 h apart.

### Laser-Induced Choroidal Neovascularization Model

Mice were anesthetized with a mixture of ketamine (100 mg/kg) and xylazine (6 mg/kg), and pupils were dilated with a combination of 0.5% tropicamide and neosynephrine-POS 5%. Corneal gel was applied to maintain hydration of the cornea and reduce media opacifications. Mice were placed in front of an argon laser (VISULAS 532s, ZEISS) after the pupils were completely dilated. A cover slip with a drop of gel was placed on the eye as a contact lens to convert the curved cornea into a planar surface. Three laser burns at equal distance from the optic disc were induced by an Argon laser with a wavelength of 532 nm, a power of 150 mW, a fixed diameter of 100 μm, and duration of 100 ms. Only burns that produced a bubble as a sign for retinal pigment epithelial rupture were included in the study. For immunohistochemical analyses, three laser burns were applied per eye. For molecular analyses (RNA sequencing and ELISA) six laser burns were applied per eye to maximize the enrichment with diseased tissue. Prior to tissue processing, the peripheral part of the retina (RNA sequencing) or choroid (ELISA) was removed with small scissors to use only the central part of the respective tissue (containing the laser lesions) for analyses. Control eyes without laser treatment were dissected in the same way. After laser treatment, mice were then placed on a pre-warmed warming plate at 35°C until they had recovered from anesthesia.

### Antibody Treatment During Laser-Induced Choroidal Neovascularization

Choroidal neovascularization was laser-induced at day 0 (d0) in *Cx3cr1*^*GFP*/+^ mice, as described above, with the following variations (Tobe et al., 1998). At d1 mice received an intravitreal injection of 50 ng anti-SPP1 (R&D Systems, AF808) solved in 1 μL PBS in one eye, whereas the same amount of IgG control antibody was injected in the same PBS volume in the contralateral eye (Nanofil Syringe 10 μl equipped with Nanofil 34 G needle, World Precision Tools, Sarasota, USA). Mice were sacrificed at d7 after performing fundus imaging and fluorescence angiography (Fluorescein (ALCON 10%, H12588-0113) was diluted 1: 20 with 0.9% NaCl and 40 μL per 20 g mouse were injected). After enucleation, eye cups were dissected to perform a collagen IV staining for the evaluation of the lesion size in the flat-mounts. Animals with subretinal bleedings or confluent lesions were excluded from further analysis. Lesion area was normalized to the size in IgG control. Normality was given using the Kolmogorov-Smirnov-Test and a ratio paired *t-*test was applied.

### Fluorescence Microscopy

After transcardial perfusion with phosphate-buffered saline (PBS), eyes were fixed in 4% PFA for 1 h at RT and processed, as previously described, for flatmounts (Pitulescu et al., 2010). Primary antibodies goat anti-mouse Collagen IV (Merck Millipore, MAB769, Burlington, USA) and rabbit anti-mouse SPP1 (LifeSpan Biosciences, LSBio, LS-B10122, Inc., Seattle, USA) were added overnight in a 1:500 (Collagen IV) or 1:1,000 (SPP1) dilution at 4°C (flatmount). Secondary antibody was applied in a dilution of 1:500 (Alexa Flour® 488 and Alexa Fluor® 647, Thermo Fisher Scientific, Waltham, USA) overnight at 4°C (flatmount). Images were taken using a conventional fluorescence microscope (Olympus BX-61 with a color camera [Olympus DP71]) (Olympus, Tokyo, Japan) and the confocal pictures were taken with a DMi-8 (Leica) with a 20x NA 0.75 CS2 (Leica 506517).

### Protein Analysis

Proteins were isolated from choroidal tissue using RIPA buffer (R0278, Sigma) containing protease (Complete Tablets Mini, 0463159001, Roche) and phosphatase inhibitors (Phosstop, 04906845001, Roche). The amount of recovered protein was evaluated by colorimetric assay (BCA kit; Pierce, Rockford, IL). Content of SPP1 (Osteopontin) in 1.25 μg of purified protein was analyzed by using an Osteopontin Mouse ELISA Kit (Thermo Fisher Scientific) according to the manufacturer's instructions.

### RNA Sequencing and Analysis

*Cx3cr1*^GFP/+^ mice were analyzed at the age of 8 weeks. For isolation of microglia, cells were stained with antibodies against CD45 (30-F11, 103133, BioLegend, San Diego, CA, USA), CD11b (M1/70, 17-0112-83, eBioscience, Thermo Fisher Scientific), Ly6C (AL-21, 45-5932-82, eBioscience, Thermo Fisher), and Ly6G (1A8, 551460, BD Pharmingen, BD Biosciences). FVD780 (eBioscience) was used to exclude dead cells. Total bulk RNA was extracted directly into RNAprotect buffer (QIAGEN, Hilden, Germany) from viable FACS-sorted CD45^lo^CD11b^+^CX3CR1^+^Ly6C^−^Ly6G^−^ retinal microglia according to the “Purification of total RNA from animal and human cells” protocol of the RNeasy Plus Micro Kit (QIAGEN). In brief, cells were stored and shipped in RNAprotect buffer at 2–8°C. After pelleting, the RNAprotect buffer was replaced by RLT Plus buffer (QIAGEN) and the samples were homogenized by vortexing for 30 s. Genomic DNA contamination was removed using gDNA Eliminator spin columns. Next, ethanol was added and the samples were applied to RNeasy MinElute spin columns followed by several wash steps. Finally, total RNA was eluted in 12 μl of nuclease free water. Purity and integrity of the RNA was assessed on the Agilent 2100 Bioanalyzer with the RNA 6000 Pico LabChip reagent set (Agilent, Palo Alto, CA, USA).

The SMARTer Ultra Low Input RNA Kit for Sequencing v4 (Clontech Laboratories, Inc., Mountain View, CA, USA) was used to generate first strand cDNA from 150 to 600 pg total-RNA. Double-stranded cDNA was amplified by LD PCR (12–14 cycles) and purified via magnetic bead clean-up. Library preparation was carried out, as described in the Illumina Nextera XT Sample Preparation Guide (Illumina, Inc., San Diego, CA, USA). Then, 150 pg of input cDNA were tagmented (tagged and fragmented) by the Nextera XT transposome. The products were purified and amplified via a limited-cycle PCR program to generate multiplexed sequencing libraries. For the PCR step 1:5 dilutions of index 1 (i7) and index 2 (i5) primers were used. The libraries were quantified using the KAPA SYBR FAST ABI Prism Library Quantification Kit (Kapa Biosystems, Inc., Woburn, MA, USA). Equimolar amounts of each library were pooled, and the pools were used for cluster generation on the cBot with the Illumina TruSeq SR Cluster Kit v3. The sequencing run was performed on an HiSeq 1000 instrument using the indexed, 50 cycles single-read (SR) protocol and the TruSeq SBS v3 Reagents according to the Illumina HiSeq 1000 System User Guide. Image analysis and base calling resulted in.bcl files, which were converted into.fastq files with the bcl2fastq v2.18 software. RNA extraction, library preparation and RNA-seq were performed at the Genomics Core Facility “KFB—Center of Excellence for Fluorescent Bioanalytics” (University of Regensburg, Regensburg, Germany).

Sequencing data were uploaded to and analyzed on the Galaxy web platform (usegalaxy.eu) (Afgan et al., 2018), as previously described (Boneva et al., 2020). Quality control was performed with FastQC Galaxy Version 0.72 (http://www.bioinformatics.babraham.ac.uk/projects/fastqc/ last access on 07/30/2020). Reads were mapped to the mouse reference genome [Gencode (Frankish et al., 2019), version M25] with RNA STAR (Dobin et al., 2013) Galaxy Version 2.7.2b (default parameters) using the Gencode main annotation file [Gencod (Frankish et al., 2019), version M25]. Two BAM files for each sample (one for each flow cell) were combined in one BAM file per sample using Merge BAM files Galaxy Version 1.2.0. Reads mapped to the mouse reference genome were counted by featureCounts Galaxy Version 1.6.4 (Liao et al., 2014) (default parameters) using the aforementioned annotation file. The output of featureCounts was imported into R Studio (Version 1.2.1335, R Version 3.5.3). Gene symbols were determined based on ENSEMBL (Yates et al., 2020) release 100 (Mouse genes, download at 08/01/2020). Genes with 0 reads in all samples were removed from the analysis. After principal component analysis (Love et al., 2014), differential gene expression was analyzed using the R package DESeq2 Version 1.22.2 (default parameters) (Love et al., 2014). Transcripts with log2fold change (log2FC) > 1.5 or < −1.5 and Benjamini-Hochberg adjusted *p* < 0.05 were considered as differentially expressed genes (DEG). Gene enrichment analysis was performed using the R package clusterProfiler 3.10.1 (Yu et al., 2012). Heatmaps were created using the R package ComplexHeatmap 1.20.0 (Gu et al., 2016). Other data visualization was performed using the ggplot2 package (Wickham, 2009). The sequencing data are available in the Gene Expression Omnibus Database under the accession number GSE160011.

### RNA Sequencing Using Massive Analysis of cDNA Ends (MACE)

Total RNA was isolated from formalin-fixed and paraffin-embedded (FFPE) sections of all specimens using the Quick-RNA FFPE Kit (Zymo Research, USA). Following a DNAse I digestion using the Baseline-ZERO kit (Epicenter, USA), the RNA concentration was measured with the Qubit RNA HS Assay Kit on a Qubit Fluorometer (Life Technologies, USA). The RNA quality was determined with the RNA Pico Sensitivity Assay on a LabChip GXII Touch (PerkinElmer, USA). The fragment size of all RNA samples ranged between 120 and 150 bp. The preparation of massive analysis of cDNA ends (MACE) libraries was carried out using 1 μg of total RNA, as previously described (Zajac et al., 2015). The barcoded libraries (four CNV membranes and four control samples) were sequenced simultaneously on the NextSeq 500 (Illumina, USA) with 1 × 75 bp. Data analysis was conducted as described above with the following modifications: Reads were mapped to the human reference genome (hg38, Galaxy built-in reference genome) with RNA STAR Galaxy Version 2.6.0b-2 6 (default parameters) using the Gencode annotation file (Gencode 31, release June 2019, downloaded on 08/05/2019, https://www.gencodegenes.org/human/releases.html). The sequencing data are available in the Gene Expression Omnibus Database under the accession number GSE146887.

### Human Tissue

Whole human eyes (ciliary body melanoma) or eye tissue (CNV membrane) were acquired after an informed consent form was signed by the patients prior to surgery at the Eye Center, University Medical Center Freiburg. At the Institute of Anatomy, eyes were enucleated in consent with the body donors, secured by contract, and no data other than age, sex, and cause of death were disclosed. MACE RNA Sequencing was performed on four FFPE-treated CNV membranes extracted from patients with neovascular AMD during vitreoretinal surgery between 1992 and 1999. Four age-matched FFPE RPE-choroidal specimens were obtained from the macular region of enucleated eyes suffering from ciliary body melanoma serving as controls. The macular RPE and choroid was unaffected in these eyes as confirmed by routine histology. FFPE tissue was sliced with 7 μm thickness and placed on glass slides. For immunofluorescence, deparaffinization (15 min xylol followed by descending ethanol concentrations with 5 min per concentration [100, 96, and 70%]) was conducted, followed by adding aqua dest at room temperature and citrate buffer (pH = 6) for 2 × 5 min at 95°C. After washing 3 × 10 min with PBS TritonX 0.3% (PBS-T), blocking solution (PBS-T, 5% goat serum) was applied for 30 min at RT. Primary antibodies (IBA1, 234 013, Synaptic Systems; mouse-anti human SPP1/Osteopontin, LS-C169155, clone 7C5H12, LifeSpan BioSciences, Inc., Seattle, USA) were diluted in PBS 0.5% bovine serum albumine (BSA) for incubation overnight at 4°C. After washing with PBS-T, slides were incubated with secondary antibodies goat anti-mouse Alexa Fluor® 647 and goat anti-rabbit Alexa Fluor® 568 for 1 h at RT. After washing with aqua dest, autofluorescence was quenched with Sudan Black B (0.3% in 70% ethanol overnight stirring at 50°C, filtrated twice and incubated for 5 min at 37°C on the slides) and nuclei were counterstained with 4′,6-Diamidin-2-phenylindol (DAPI) 1:10,000 for 10 min, washed three times with aqua dest, and embedded in Fluorescence Mounting Medium (Agilent Dako).

### Statistical Analysis

Statistical analysis was performed using GraphPad Prism (GraphPad Software, Version 6.0, La Jolla, USA). Data were tested for normality applying the Kolmogorov–Smirnov test. If normality was given, an unpaired *t-*test was applied, if not indicated otherwise. If the data did not meet the criteria of normality, the Mann–Whitney test was applied. Differences were considered significant when *P* < 0.05.

## Data Availability Statement

The original contributions presented in the study are publicly available. This data can be found at: https://www.ncbi.nlm.nih.gov/geo/query/acc.cgi?acc=GSE160011.

## Ethics Statement

The studies involving human participants were reviewed and approved by Ethics committee of the University of Freiburg. The patients/participants provided their written informed consent to participate in this study. The animal study was reviewed and approved by Regierungspräsidium Freiburg i. Br.

## Author Contributions

AS, PZ, AT, D-DR, and PW conducted experiments and analyzed data. SB provided CNV tissue and human RNA-seq data. JW analyzed bulk RNA-seq data. PW wrote the manuscript with support from AS, SB, GS, and CL. CL and PW supervised the project. All authors contributed to the article and approved the submitted version.

## Conflict of Interest

The authors declare that the research was conducted in the absence of any commercial or financial relationships that could be construed as a potential conflict of interest.
